# Integrating Spirituality and Religious Beliefs in a Mindfulness Based Cognitive Behavioral Therapy for PTSD with Latinx Unaccompanied Immigrant Children

**DOI:** 10.1007/s40653-023-00541-1

**Published:** 2023-04-21

**Authors:** Lisa R. Fortuna, William Martinez, Michelle V. Porche

**Affiliations:** Department of Psychiatry and Behavioral Sciences, University of California San Francisco, Zuckerberg San Francisco General Hospital, San Francisco, CA 94110 USA

**Keywords:** Unaccompanied Immigrant Children, Traumatic Stress, Mindfulness, Cognitive Therapy, Religiosity, Spirituality

## Abstract

**Purpose:** Unaccompanied immigrant children (UIC) experience significant mental health concerns, particularly posttraumatic stress. This is a vulnerable population, yet little systematic research has examined the effectiveness of evidence-based models such as cognitive behavioral therapy (CBT) to meet their needs. Integrating religious beliefs and spirituality into therapy could elucidate better understandings of traumatic stress, and posttraumatic cognitions when working with UIC with strong faith traditions/beliefs. **Methods:** We report on modifications made to a pre-existing treatment, consisting of integrating religious and spiritual themes, to engage and work with UIC participants in a pilot study of Mindfulness-Based CBT. Thematic analysis of therapy notes evaluated the implementation process for integrating religious and spiritual themes. Three composite vignettes illustrate how religiosity and spirituality were salient for UIC participants in this pilot study, and how these were integrated into therapy to address posttraumatic cognitions and symptoms. We assessed changes in PTSD symptom severity and posttraumatic cognitions for UIC and in comparison, to non-UIC participants using the Child PTSD Symptom Scale and the Posttraumatic Cognitions Inventory. **Results:** Religiosity and spirituality were important for coping and conceptualizations of trauma, served as facilitators for engaging UIC in therapy, and related to improving posttraumatic cognitions and symptoms. **Conclusion:** Religious identity and spirituality can be important for meaning making, trauma cognitions and symptoms, and can be important to explore in therapy with unaccompanied immigrant children and adolescents. **Clinical Trial Registration:** Not applicable.

## Introduction

Humberto (Pseudonym), 16-years-old, had a hard time when he arrived in the United States. He is an unaccompanied immigrant minor who arrived at the United States border after running away and seeking safety from gang violence in El Salvador. He shared a story with his therapist about an event that happened when he was in his country. It was about a time when he was peddling his bike as fast as he could because gang members were chasing him and shooting at him. They had wanted him to join them, and he had refused. He recounts that the bullets miraculously missed him. He believes a divine protection must have been involved. He sometimes still has nightmares about that event, but he believes God may have a purpose for him. After all, God saved him. Humberto’s grandparents taught him all he knows and believes about God. His faith is what gave him the courage to refuse recruitment by gangs. His faith taught him that you are good and kind towards people. You do not kill or harm others like gang members do. His faith is also why he has feelings of guilt and regretful thoughts about having left his country and his family behind.

At the end of 2021, 89.3 million people worldwide were forcibly displaced from their homes, 41% of whom were children under the age of 18 (UNHCR, 2021). In 2019, the U.S. apprehended over 73,000 unaccompanied immigrant children at the U.S.-Mexico border, surpassing the previous high set in 2014 (U.S. Customs and Border Protection, 2020). The sociopolitical and sociocultural contexts of individuals’ countries of origin contribute significantly to decisions to migrate or flee (Portes & Rumbaut, 2014). Families may send their children ahead of adults and unaccompanied in the hopes they will find safety in the U.S., which eludes them at home (UNHCR, 2014). Unaccompanied immigrant children (UIC), like Humberto experience traumatic stress pre-migration, en route, and post-migration (Seghetti et al., 2014). For example, UICs may be exposed to physical and sexual violence, be separated from family members, or experience barriers to basic needs such as food and shelter, all before they are apprehended at the U.S.-Mexico border (UNHCR, 2014). Once young migrants arrive on U.S. soil, they also face the detrimental impacts of racism and discrimination, all while needing to rapidly adapt to a new environment (Chavez-Dueñas et al., 2019). The cumulative traumas and stressors UIC experience, place them at risk for poor mental health including posttraumatic stress disorders (PTSD), anxiety, and depression (von Werthern et al., 2019).

Spirituality, religious beliefs, and faith have been found to be important for coping among immigrant children (Raghallaigh, 2011; van Es et al., 2019), and can be incorporated into evidence-based care, such as cognitive behavioral therapy (CBT) (Cervantes, 2019). When treating children and adolescents, it is increasingly important for mental health care professionals to consider how religious identity or spirituality, can influence meaning making, perception of distress, and coping (Aggarwal et al., 2016; Sexson, 2004). A meta-analytic review found that religious involvement is more strongly associated with prosocial behaviors than behavioral problems (Cheung & Yeung, 2011) for youth from diverse backgrounds. Religious identity can moderate the relationship between hope and anxiety symptoms for Latinx youth of Mexican heritage, whereby increased religious identity and increased hope are associated with less anxiety (DiPierro et al., 2018). A multinational study in Canada and England (non UIC) found positive mental health outcomes associated with spirituality, and in particular, the domain of spirituality was a mediator of positive connection to self (Michaelson et al., 2019). A review of spiritual or religious coping and psychological trauma, although focused mostly on adult survivors of trauma, suggested that religious coping is helpful for trauma recovery (Bryant-Davis & Wong, 2013).

## Mindfulness-Based Cognitive Therapy with Unaccompanied Immigrant Children

Existing evidence-based treatments are helpful for immigrant children with depression, anxiety, and PTSD (Pineros-Leano et al., 2017; Sullivan & Simonson, 2016; Tyrer & Fazel, 2014). These include various forms of cognitive behavioral therapy (e.g., Kataoka et al., 2003), narrative therapy (e.g., Ruf et al., 2010), and creative expression (Sullivan & Simonson, 2016). However, to our knowledge, no studies have examined the role of religiosity or spiritualty within evidence-based therapy interventions. Mindfulness-Based Cognitive Therapy for Adolescent PTSD and Addictions (MBCT-Dual), the guiding framework for the therapy model (Fortuna et al., 2018; Fortuna et al., 2015) and study we report on here, shown to be effective for reducing PTSD and depression symptoms in traumatized youth, is based upon the Cognitive Model of Trauma (Ehlers & Clark, 2000). The Cognitive Model of Trauma proposes that two key processes lead to a sense of ongoing and current threat that serves to maintain PTSD symptoms: (1) individual differences in the appraisal of the trauma and/or its sequelae, and (2) individual differences in the nature of the memory for the event and its link to other autobiographical memories and beliefs which in turn influences the interpretation of current situations (Ehlers & Clark, 2000). Given this framework, cognitions and appraisals about trauma that are informed by religious or spiritual beliefs may be particularly important to consider for individuals from highly religious cultures and contexts (Kucharska, 2018).

MBCT-Dual (see Table 1) helps participants learn to use mindfulness practices to increase awareness to feelings, facilitate self-compassion, and identify patterns in thinking that contribute to distress and risky behaviors including drug use, and to practice emotional regulation (Fortuna et al., 2020). It is a manualized, individual 12-week therapy. The central components of the therapy are mindfulness and cognitive restructuring, which when combined help youth identify and re-evaluate trauma influenced thinking patterns, manage mood states, and automatic reactions.

**Table 1:** Sequence of therapy sessions

	Therapy Sessions
Meeting 1	Introduction, Engagement, and Safety Plan
Meeting 2	Introduction to Mindfulness Practice
Meeting 3	Mindfulness Practice, Psychoeducation 1: PTSD, Trauma Cognitions and Automatic Thinking.
Meeting 4	Mindfulness Practice, Psychoeducation 2: Risk Behaviors, Drug and Alcohol Triggers
Meeting 5	Managing Triggers, Cravings, and Urges
Meeting 6	Mindfulness Practice, Common Styles of Thinking, and cognitive reappraisal
Meeting 7	Observing our Thoughts and Cognitive Restructuring
Meeting 8–11	Mindfulness Practice and Cognitive Restructuring Practice, Practicing Recovery Skills
Meeting 12	Ending Therapy and Relapse Planning

The first author conducted a clinical trial for MBCT-Dual that included thirty-seven adolescents, with co-occurring PTSD and substance use problems, were either English or Spanish dominant, including 11 immigrant youth (6 who were UIC), and youth experiencing multiple traumas and psychosocial stressors. 62% (62%), including all six UICs, were study completers as defined by retention for at least 6 weeks of treatment. There were significant improvements in PTSD and depression symptoms from baseline to end of treatment, reflecting medium effect sizes. Improvements in PTSD symptoms were related to changes in trauma-associated cognitions. We found that UIC study participants frequently expressed religious themes that were associated with their posttraumatic cognitions and strategies for coping. This led us to consider how to integrate religiosity and spirituality, and related cognitions and beliefs into MBCT-Dual more intentionally and to be responsive to their experiences.

Through a mixed methods study approach of UIC participants in the MBCT-Dual pilot study, this paper provides insights into how we made modifications in the therapy by integrating the religious and spiritual themes, especially in helping participants reframe posttraumatic cognitions and enhance coping. We focus on exploratory analysis of the modifications made to MBCT-DUAL in using spiritual/religious topics, and implications for the outcomes of the MBCT-Dual therapy (specifically effects on posttraumatic cognitions and symptoms) for UIC study participants, and relative to other study participants.

## Methods

In a pilot clinical trial of Mindfulness-Based Cognitive Therapy-Dual (MBCT-Dual), we used a sequential mixed method approach (Creswell & Plano Clark, 2017) to quantitatively evaluate changes in PTSD, depression and anxiety symptom severity, and posttraumatic cognitions, and to capture qualitative meanings of trauma and trauma related cognitions shared by youth. Given the dearth of therapy research conducted specifically with UIC we evaluated whether there was any difference in change in symptoms and posttraumatic cognitions among unaccompanied immigrant children as compared to United States (U.S.) born youth in the study, using standardized measures. Then, using thematic coding and analysis of study therapy case notes and records we explored themes related to trauma cognitions and coping that emerged in therapy. Based on these results, we constructed three composite and de-identified case study vignettes to present how the integration of religious/spiritual themes related to addressing posttraumatic cognitions for UIC participants.

### Intervention Development

Mindfulness-Based Cognitive Therapy-Dual (MBCT-Dual) is a manualized, 12-week, mindfulness-based cognitive behavioral therapy adapted for co-occurring PTSD and substance use disorders by the first author from a model developed for PTSD by Drs. Mueser and Rosenberg (2008) and their research team. The manualized therapy integrates cognitive behavioral therapy, mindfulness, and recovery skills in each session and specifically: (1) psychoeducation about trauma and the nature of thoughts; (2) relaxation and breathing retraining; and (3) mindfulness practices for improving awareness of thoughts, feelings, and reactions, and for considering new ways of responding. Table 1 provides descriptions of each therapy session. Individuals are guided through regular mindfulness practices and a five-step cognitive restructuring approach in which they identify stressful/triggering situations, identify associated thoughts and feelings, weigh the evidence for their thinking, consider reappraising their thinking, and finally create a helpful action plan for addressing or coping with ongoing stressful situations. The action plan and coping strategies are chosen by the participants with the support of their therapist and can include the implementation of coping strategies like spirituality. The therapy model reinforces therapist self-practice of mindfulness, which is important for guiding youth in their practice of mindfulness.

### Considerations for Integrating Religiosity/ Spirituality into MBCT-Dual

Some basic principles emerged for integrating spirituality in MBCT-Dual that stem from best practices for engaging adolescents in trauma therapy and using active mindful listening by the clinician. The objective was always to give participants the advantage of seeing things in a new light and to help them increase their capacity to recognize their own mental events. This included:

1. Starting with mindfulness practices that are less trauma triggering, e.g., mindfulness walking or mindfulness eating, as compared to starting with a Loving Kindness meditation, which requires more advanced self-compassion and readiness for becoming aware of potentially stressful thoughts. Starting with simpler and more accessible practices contributes to creating a psychologically safe space for building trust and supporting youth through the vulnerability of engaging in trauma-focused therapy and mindfulness practice. Youth are progressively guided in approaching their thoughts with curiosity and as an opportunity for responding in new ways.
2. Asking permission from the youth to explore the religious/spiritual themes that emerge, with openness to the outcome of that discussion and with curiosity.
3. Being aware that religious and spiritual themes can inform both negative and positive cognitions about the world, self, and others and be open to exploring this with youth. For example, a youth may find comfort in what they perceive as a loving God. In contrast, youth may feel they are divinely judged or unworthy of God’s love because they have done bad things. The therapy can then be informed by these emerging religious/spiritual themes, and with an understanding for how they can serve as adaptive coping or perpetuate posttraumatic cognitions like guilt and shame, and negative images of self.
4. Acting with spiritual-cultural humility is important. The point is not to impose or assert a religious or spiritual perspective but to show an openness to learning together about the meaning of spirituality, religiosity, and faith beliefs and how they are experienced and understood by the youth.
5. Exploring how religious and spiritual themes emerge for youth and relate to their sense of self- agency for coping and healing. For example, if they use prayer, how do they use it to help with distress?
6. Identifying and working simultaneously with other posttraumatic cognitions and themes that may not be directly related to spirituality or religiosity. Not everything is related to religiosity and spirituality; however, considering these invites an opportunity to include an aspect of human healing and well-being often overlooked in traditional therapy.

The MBCT-Dual pilot study was conducted by the first author and masters-level clinicians (all bilingual/bicultural), using a structured therapy manual that was culturally adapted with input from youth, including immigrant youth through focus groups that included immigrant youth. Their recommendations for cultural adaptations of the study therapy during its development (see Fortuna, 2018) included the importance of asking about family relationships, migration stressors, cultural understanding of problems and asking about religion and/or spirituality or other related practices that are important for a participant’s coping. Focus group informants also suggested appropriate words in Spanish to discuss concepts like mindfulness (i.e., la atención plena) and trauma (i.e., adversidad). To monitor treatment fidelity, the first author and two trained research assistants listened to all recorded therapy sessions and evaluated these using a fidelity checklist.

### Recruitment and Sample

Human subjects’ approval was obtained by the first author’s Institutional Review Board. Informed consent for research participation was obtained from the legal guardians of all participants. Participants in the MBCT-Dual pilot clinical trial were recruited from community-based and school-based clinics serving primarily low-income or publicly insured patients in Central Massachusetts, through clinician or case manager referrals and study flyers. Thirty-seven adolescents participated in a pilot study; six were the Spanish language dominant unaccompanied immigrant children that are the focal children in this current study. All UIC participants were referred by an Unaccompanied Refugee Minors Resettlement Program in Central Massachusetts which, in accordance with best practices (Crea et al., 2018), provides culturally and linguistically appropriate child welfare, foster care, and independent living support services to children who do not have parents or other legal guardians/caregiver sponsors in the U.S., or who enter the U.S. unaccompanied by a parent, an immediate adult relative, or an adult having documentable legal evidence of custody of the minor. All the children were living in foster care settings and were applicants for special juvenile status or asylum. One girl and two boys came from Guatemala, one boy from Honduras, one boy from El Salvador, and one boy from Mexico; the mean age for this subsample was 15.8 years; all treatment and data collection was conducted in Spanish. One of the boys from Guatemala identified as indigenous, his first language was K’iche and he was also fluent in Spanish. This small qualitative sample reflects principles suggested by information power guides (Malterud et al., 2016) for design that includes a narrow research aim. In this case, the specificity of the focal group of unaccompanied immigrant children allowed for an exploratory thematic analysis, with data gathered by a bilingual and bicultural child psychiatrist with an established history working with this population. For statistical comparison of outcome measures, we included the U.S.-born children from the original sample who completed full treatment and pretest and posttest measures in English (*n* = 14). For further details on the full sample and parent study see Fortuna et al. (2018).

### Measures

All study measures were completed at intake (baseline) and end of treatment by a trained research assistant, a native Spanish speaker who was not involved in the delivery of the therapy. All clinical measures have been translated and validated for use in Spanish except the Upsetting Events Survey (UES). The UES translations was completed using forward and back translation by the Spanish language translators. A standardized demographic sheet was designed for the study to collect sex, age, race/ethnicity, parental education, preferred language (English/Spanish), number of siblings, and country of birth. Adolescents were screened for potential eligibility using the *PTSD Checklist-Civilian Version* (PCL‐C; (Blanchard et al., 1996) with a cut‐off total score for study inclusion of 36 (score cut‐off recommended for civilian populations with estimated prevalence of PTSD 16–39%). The PCL‐C (civilian) is a 17‐item self‐report screening measure for DSM‐IV PTSD, which presupposes exposure to a Criterion A traumatic event.

### Traumatic Experiences and PTSD

#### Traumatic exposures

Exposure to traumatic events were documented using the Upsetting Events Survey (UES) (Rosenberg et al., 2014) which includes questions on past exposures to traumatic experiences (e.g., physical injury, experiencing and witnessing violence, disasters, sexual abuse/assault). The UES also allows for entry of other traumatic experiences not asked by the measure.

#### PTSD

Child PTSD Symptom Scale: Baseline and change in PTSD symptom severity was assessed using the Child PTSD Symptom Scale (CPSS) (Foa, 2001). The CPSS is a 26-item self-report measure that assesses PTSD diagnostic criteria and symptom severity in children ages 8 to 18. The CPSS yields a total symptom severity scale score (ranging from 0 to 51; α = 0.87) and a total severity-of-impairment score (ranging from 0 to 7; α = 0.72). Scores can also be calculated for each of the three PTSD symptom clusters of re-experiencing (α = 0.79), avoidance (α = 0.70), and hyperarousal (α = 0.67) (Foa, 2001).

#### Posttraumatic cognitions

The Posttraumatic Cognitions Inventory (PTCI; Foa et al., 1999) is a validated 33-item measure of trauma-related thoughts and beliefs that assesses negative cognitions associated with PTSD including self-blame, negative beliefs about self, and negative beliefs about the world. Examples of PTCI items include: *Nothing good can happen to me anymore* (self), *I will not be able to control my anger and will do something horrible* (self), *I can’t rely on other people* (world), *you never know when something bad will happen* (world), *what happened was because of the kind of person I am* (blame) and *I should have done something to prevent what happened* (blame). Individuals rate the extent to which they agree or disagree with each statement on a 7-point scale (1 = totally disagree,7 = totally agree). A total score (α = 0.95) was calculated as the sum of the three subscales (Negative Cognitions About the Self, α = 0.93; Negative Cognitions About the World, α = 0.81; Self-Blame, α = 0.74). In a study of automotive accident survivors (Beck et al., 2004), test–retest reliability for 1-week interval ranged from 0.75 to 0.89 and for a 3-week interval ranged from 0.80 to 0.86 for the three subscales. All three PTCI scales, as well as the total score, have been found to be correlated substantially with severity of PTSD, depression, and general anxiety symptoms. Discriminant function analyses were performed to test how accurately individuals with PTSD could be identified within an overall sample of trauma survivors. The three PTCI scales classified 86% of the traumatized individuals correctly into those with and without PTSD, Wilks’ A = 0.47, v2 (3, N = 355) = 259.07, p < .0001 (Foa et al., 1999).

### Qualitative Data

Qualitative data for thematic case study analysis were collected from several study sources including detailed therapy session notes and brief study therapy notes, as well as participant response to the open-ended questions on the UES, CPSS, and end of treatment interviews. Because of agreements for protection of unaccompanied immigrant children, we were limited to analysis of notes contained in charts, as no audio recording was allowed during sessions. The first author conducted all the therapy sessions for the Spanish speaking participants, wrote all therapy notes and translated any items which were in Spanish into English for this analysis. Case history summaries were developed for each of the unaccompanied child participants and included information about reasons for leaving their country of origin and stressors before, during, and after migration. The coding was conducted from case histories in addition to clinical notes.

#### Analysis

Descriptive and inferential statistics were conducted, including one-tailed paired *t*-tests to assess hypothesized improvement in outcomes comparing unaccompanied and U.S.-born participants; Cohen’s *d* was calculated to measure differences in effect sizes for the two groups. Two independent coders (the first two authors, both Latinx, one a child psychiatrist and the other a child psychologist) used semantic thematic analysis (Braun & Clarke, 2006) to code the clinical notes of children in treatment. Using Braun and Clarke’s (2006) thematic coding process, authors read through all available qualitative data, then generated three a priori codes that were developed in alignment with the three subscales of the PTCI: Negative Cognitions of the Self, Negative Cognitions about the World, and Self-Blame. For data that did not correspond to these three PTCI codes, authors used open coding to break up the material into discrete and meaningful parts. From this process, authors identified one additional code reflecting adaptive coping of Religion/Spirituality. Data was organized into identified themes across data sources using NVivo and then output was reviewed, further defining and refining themes. Coding disagreements were resolved through consensus after the third author reviewed coded session notes.

## Results

### Quantitative Results: Reports of Trauma Exposure

The most common traumas reported in the U.S.-born English dominant subsample (*n* = 14) included witnessing domestic violence, sexual abuse, physical abuse, loss, or injury; the subsample was comprised of 10 boys and 4 girls, 5 Latinx and 9 White, mean age of 16.2. The UIC subsample was comprised of six patients, one girl and five boys; the mean age was 15.8. For the UIC, five of the children reported physical assault or abuse as their primary trauma and the sixth child reported the witnessing of his mother being murdered by a gang in his home country. The UIC subsample reported an average of seven traumatic experiences on the UES. The U.S.-born children in the sample reported an average of six traumatic experiences.

### Quantitative Results: Treatment Outcomes for U.S.-Born Subsample vs. UIC Subsample

The overall sample from the parent study showed significant improvement in PTSD symptoms and change in cognitions (Fortuna et al., 2018). All six UIC completed both treatment and full assessment battery (Table 2) compared to just over half of the 27 U.S.-born youth. The U.S.-born subsample showed significant improvement in PTSD symptoms (*t* = 1.80, *p* < .05), combined PTCI cognitions (*t* = 2.51, *p* < .01) and Negative Cognitions about the Self (*t* = 3.25, *p* < .01). Comparative tests for the UIC subsample showed similar directionality of results for reduction in PTSD symptoms (*t* = 2.14, *p* < .05). Tests for change in cognitions overall also showed significant improvement (*t* = 2.27, *p* < .05), whereas there was significant improvement on the Negative Cognitions about the World subscale (*t* = 2.52, *p* < .05), improvement in Negative Cognitions about the Self approached significance (*t* = 1.51, *p* < .10), and no statistical difference in change the Self-Blame subscale.

**Table 2:** Treatment Comparisons: Unaccompanied Immigrant Children vs. U.S.-Born English Dominant Children

	Unaccompanied (n = 6)				U.S.-Born (n = 14)				
	Baseline [95% CI] (s.d.)	Time 2 [95% CI] (s.d.)	Cohen’s *d*		Baseline [95% CI] (s.d.)	Time 2 [95% CI] (s.d.)	Cohen’s *d*		
CPSS Total	21.67 [10.09, 33.25] (14.47)	8.33* [2.03, 14.63] (7.87)	1.15		26.36 [19.96, 32,76] (12.21)	22.50* [16.87, 28.13] (10.75)	0.34		
PTCI Combined	115.67 [71.69, 159.65] (54.97)	87.67* [58.91, 116.43] (35.94)	0.60		139.07 [114.47, 163.67] (46.96)	120.64* [104.89, 136.39] (30.06)	0.47		
PTCI Self	54.67 [26.56, 82.78] (35.13)	40.83~ [25.37, 56.29] (19.32)	0.49		77.21 [61.12, 93.30] (30.71)	64.43** [52.64, 76.22] (22.51)	0.47		
PTCI World	42.17 [30.62, 53.72] (14.44)	30.83* [20.28, 41.38] (13.18)	0.82		41.86 [35.77, 47.95] (11.63)	39.50 [33.98, 45.02] (10.54)	0.21		
PTCI Blame	18.83 [9.74, 27.92] (11.36)	16.00 [7.82, 24.18] (10.22)	0.26		20.00 [15.36, 24.64] (8.86)	16.57~ [13.44, 19.70] (5.97)	0.54		

~ *p* < .10; *p < .05; **p < .01

### Qualitative Results: Themes Related to Context of Cognitions and Adaptive Coping

The following results describe how unaccompanied immigrant children reported on negative cognitions about self, negative cognitions about the world, and self-blame. A priori coding was used to enhance understanding of UIC individual change scores on the PTCI subscales (Table 3). The additional code of religion/spirituality as a coping response suggested a possible facilitator, or barrier, for treatment. We reviewed the data to check for religion and spirituality as an adaptive coping response theme for non UIC participants and found none.

**Table 3:** Individual change in symptoms for unaccompanied immigrant children

ID	CPSS Total	PTCI Combined	PCTI Negative Cognitions of Self	PCTI Negative Cognitions about the World	PCTI Self-Blame
1001	Pre: 36 Post: 20 Change = -16	Pre: 215 Post: 154 Change = -61	Pre: 121 Post: 69 Change = -52	Pre: 56 Post: 51 Change = -5	Pre: 38 Post: 34 Change = -4
1004	Pre: 7 Post: 1 Change = -6	Pre: 112 Post: 56 Change = -56	Pre: 43 Post: 21 Change = -22	Pre: 43 Post: 29 Change = -14	Pre: 26 Post: 6 Change = -20
1005	Pre: 19 Post: 14 Change = -5	Pre: 100 Post: 64 Change = -36	Pre: 45 Post: 26 Change = -19	Pre: 41 Post: 28 Change = -13	Pre: 14 Post: 10 Change = -4
1007	Pre: 21 Post: 2 Change = -19	Pre: 54 Post: 70 Change = + 16	Pre: 33 Post: 37 Change = + 4	Pre: 15 Post: 15 Change = 0	Pre: 6 Post: 18 Change = + 12
1017	Pre: 41 Post: 2 Change = -39	Pre: 129 Post: 100 Change = -29	Pre: 63 Post: 60 Change = -3	Pre: 52 Post: 21 Change = -31	Pre: 14 Post: 19 Change = + 5
1025	Pre: 6 Post: 11 Change = + 5	Pre: 84 Post: 82 Change = -2	Pre: 23 Post: 32 Change = + 9	Pre: 46 Post: 41 Change = -5	Pre: 15 Post: 9 Change = -6

Note: Shaded cells show increase in symptoms and cognition

1001: Adriana; 1005: Ricky; 1007: Martin (vignette pseudonyms)

### Expressed Cognitions Aligned with PCTI Subscales

The Posttraumatic Cognitions Inventory (PTCI; Foa et al., 1999) is composed of three subscales: Negative Cognitions About the Self, Negative Cognitions About the World, and Self- Blame. Each of these subscales provides information on important appraisals that can emerge because of traumatic experiences. The following are some examples of the ways that these cognitions emerged thematically for unaccompanied immigrant minors in the study.

#### ***Negative cognitions about self***

This PCTI subscale includes items that reflect the construct of not trusting one-self or that one is damaged or unreliable (items on the subscales include *I feel like I do not know myself anymore, I feel dead inside, I can’t rely on myself, I can’t deal with the slightest upset*). Narrative examples from coded notes were found for four of the UIC and reflected the children’s feeling incapable of having “control of my life” and feeling unable to control emotions: “I am going to lose control and fight.” They also expressed doubts about being able to succeed at school or with their family relationships.

#### ***Negative cognitions about the world***

These items of the PCTI subscale capture fears and mistrust about the world and people that have resulted from their experiences (*I can’t trust others, the world is unsafe, you never know when something bad is going to happen, people are not what they seem*). Relevant codes were found in the case histories and clinical notes. Five of the six UIC children described examples of mistrust and feeling unsafe. Specifically, they named “foster homes” as unsafe spaces. They described needing to stay to themselves, distress about harmful conflicts in relationships “that caused” them to need therapy, feeling misunderstood, and isolated. They described their responses to, and their thinking about, an unjust, unsafe world. This was particularly challenging for UIC dependent on new adults in their lives. As one youth reported, “I did not trust my caseworker, I felt vulnerable.” As one other boy described, he “often feels isolated” because he can’t even trust his one friend.

#### ***Self-blame***

The items for this PCTI subscale are also cognitions about self but more specifically reflect blaming oneself for experiencing traumatic events (*the event happened because of the way I acted, someone else would have gotten out of the situation, someone else would have stopped the event from happening*). Coding of self-blame was described by three of the UIC and included narratives about blame, needing forgiveness, guilt about what happened or for decisions they made. A female participant from Guatemala talked about believing her father’s accusations that she is “irresponsible” and that “she will never change” and that because of her deficits she is to blame for her family’s troubles. She went on to further describe “feeling guilt about doing well in school” here in the US while her family “struggled with money and safety problems” in her country of origin. In this latter issue, self-blame and guilt about the trauma became generalized to other subsequent situations and stressors.

### Spirituality/Religiosity as Adaptive Coping Theme

Themes related to coping strategies or adaptive coping come primarily from cognitive restructuring sessions and, more specifically, from action plan discussions. In these discussions, participants explore how to address a problem or how to cope with a situation if either (1) their thought remained true even after challenging or reframing it using cognitive restructuring, or (2) the stressful triggering situation warranted a plan, solution, or adaptation. Spirituality and religiosity were common themes, particularly salient for the UIC participants in the study and presented in various ways. Mentions of religious or spiritual coping also offered insights into how UIC used these beliefs as an explanatory model for their trauma and interpretation of events, and also influenced posttraumatic cognitions and recovery.

Five of the UIC described religiosity or spirituality as providing an important way to cope with trauma experiences and the challenges of migration. A girl from Guatemala, first experiencing guilt about her boyfriend’s murder, later interpreted being “forgiven” by him through reconciliation she attained by speaking with his spirit and his becoming a “guardian spirit” for her (healing from guilt and self-blame). A boy from El Salvador talked about more formal aspects of religion, including being sustained by “a star that comes from God’s heart to shine over everyone” and that he once experienced chest pain at church while “thinking about the bad things he had done in the past.” One of his most transformative cognitive reframings became “God has a purpose for everyone including me.” A boy from Mexico described the importance of prayer in “helping him calm down and cope” and went on to say that he “used prayer to stay connected to family back home by praying for their protection and sustenance.” This in turn helped him develop a more positive self-regard and sense of agency.

Religious/spiritual themes influenced both negative cognitions about self (e.g., *I am bad and damned)* and about the world *(the world is evil*) as well as positive cognitions (e.g., *I am protected, I am forgiven*). For example, a boy from Honduras had drawn pictures of the devil killing people while he was in an immigrant/refugee resettlement center for children. He had been asked about the meaning of the drawing by a counselor at the center, but he had dismissed its importance and had refused to talk about it. The sense of vulnerability of being in an immigration center may have contributed to his hesitancy to explore the drawing. Later, after the creation of a therapeutic alliance and sense of safety with his therapist in the community he was able to talk about the meanings of his drawings. With an expressed curiosity and respect for discussing spiritual and religious themes by his therapist, the boy was able to talk about how he had thought he was a bad person when he was at the detention center. Back then, he thought his ability to be destructive was stronger than his ability to do “good things,” and no one could help him change that about himself (cognitions about self). The drawing of the devil was a way for him to express his anger. He also admitted that he had been treated so badly in his life that maybe he was a demon deserving of all that poor treatment, and that he was to blame (self-blame). This is one example of how religious themes served to sustain negative posttraumatic cognitions and expressions about self and others that needed to be explored.

Therefore, an integration, or at minimum, an exploration of spiritual-religious themes as part of MBCT-Dual presented a therapeutic opportunity for integrating these into a trauma treatment and especially in working with trauma related cognitions. Spiritual-religious themes were also intersectional with cultural beliefs such as beliefs in the existence of spirits, divine intervention and protections, and fatalism, the reliance on God’s will and belief that events are fixed by God in advance and human beings are powerless to change it. In turn, these spiritual-religious themes had relevance in influencing how young people understood the intentions of others, and the beneficence or maleficence of the world and self. These in turn influenced cognitions about traumatic experiences. That is, religion and spirituality beliefs intersected with traumatic experiences to influence cognitions about the world, others and self as well as contributed to coping. The following composite clinical examples illustrate how spiritual-religious themes were integrated into the MBCT-Dual model when working with the unaccompanied immigrant children in this study, including in exploring posttraumatic cognitions and coping. Pseudonyms are used and several other details changed to deidentify the cases and maintain confidentiality.

### Vignettes illustrating the integration of spiritual-religious themes

As mental health practitioners, we can borrow from ethnographic approaches to inform our understanding of trauma and its narrative account in the context of culture and spirituality, including the meaning of visions or beliefs about encounters with ghosts and spirits (Goulet, 1998). The following is a composite vignette where spirituality played an important role in exploring and reconciling a traumatic past relationship and posttraumatic cognitions associated with guilt and shame. The vignette illustrates the careful progression from engaging in mindfulness practice, to observing when the youth began to feel comfortable sharing posttraumatic cognitions and encounters with the spiritual world as sessions progressed.

### ***Vignette 1: Spirits, Visions, and the spiritual world of trauma***

Adriana (a pseudonym) was a 15-year-old Spanish-speaking unaccompanied female participant from Central America. She had experienced physical and psychological abuse in her country of origin and was a victim of sexual assault and torture from gang members. Her father was alcohol dependent and was physically abusive towards her mother, Adriana, and her younger brother. Adriana had moderate depression and severe PTSD symptoms at the beginning of therapy. Her primary PTSD cognitions related to her inability to trust others, viewing the world as unsafe and thinking that what happened to her was a result of what kind of person she is. She had difficulty focusing on school because she experienced frequent flashbacks and trauma memories about assaults during the day and nightmares kept her up at night. The therapist first introduced her to mindfulness practice by utilizing her breath as the focus, and mindful walking to help her be more present and aware of her thoughts and body sensations in the moment. A focus was on helping Adriana understand how her thoughts and emotions are impermanent and how this offered an opportunity for expanding her awareness of what she was experiencing in the moment. At first, she was only mildly interested in mindfulness practices. However, she found that listening mindfully to music (e.g., slow Spanish songs) and ambient sounds helped her relax and to focus. For some weeks she preferred to keep herself busy and to not “think too much.” Her depression symptoms persisted after several weeks of therapy and despite several cycles of practicing cognitive restructuring.

However, during one session, after 5-minutes of mindfulness practice with sounds—she shared that she was aware of thoughts and a desire to speak to her mother who was still in her country of origin. Adriana reported that she was feeling guilty about the bad things that had happened between her and her mother. She also felt disappointed that no one had helped her when she had most needed it. Not only that, in her own mind she was a bad person and to blame for bad things that happened (self-blame), for associating with gang members and for not listening to her mother’s warnings of dangers. Adriana disclosed that her stressful feelings and thoughts “often come out” in her dreams. She told of a recurrent dream in which she walks into a circular body of water while a group of people surrounded her on the shoreline doing nothing more than watching her. After walking into the water, she began to change her mind about being there—she felt painful regret and shame. Gentle questions framed with curiosity by the therapist about what Adriana thought this dream meant revealed her spiritual interpretation of the dream. First, the people standing around the body of water were both spiritual ancestors and living elders. From her spiritual tradition, it is important to have the blessing and support of these elders and to honor them. She expressed shame and felt disconnected from them because of what she had done badly and because of her poor choices.

Adriana agreed to try cognitive restructuring by using the experience with the elders—ancestors in the dream—as the stressful situation. Cognitions that she could identify from exploring the dream related to past traumatic situations (i.e., doing regrettable dangerous things and no one helping her through the decisions or the consequences). She was able to identify the related trauma cognition, as paraphrased here: *I can’t trust anyone*, *the events that happened to me were because of the sort of person I am, I can’t forgive myself, I am not forgivable*. She identified that she regretted her actions and blamed only herself (not mainly the individuals who assaulted her) for the violence she had endured (self-blame). In deciding which related cognitions she wanted to work on, she identified: “bad things happen because of the type of person I am”. She then shared that she believed she may even be a sinful person (cognitions about self) and that neither God, ancestors nor community could forgive her. The following outlines how cognitive restructuring was implemented in using the dream as the stressful situation:

Step 1—The Event: Her recurrent dream in which she walks into a body of water and a group of people (elders and ancestors) are watching her. After she is in the water, she changes her mind.

Step 2: Practice mindfully sitting with that experience and identifying her thoughts with curiosity and about any associated feelings: which were guilt, shame, anger, disconnection/isolation (from elders, from community)

Step 3: Thoughts: I cannot forgive myself. I am a bad person. I am wrong and shameful, even sinful (cognitions about self) and I am to blame (self-blame).

Step 4: Challenging or reframing thoughts: From the mindfulness perspective this was an opportunity to explore thoughts as informative although not necessarily factual. She was also offered an opportunity to expand her awareness of what she was experiencing in the moment. Using the cognitive reframing step, she weighed the evidence of whether her thoughts were valid: Adriana continued to find “evidence” to support her thought that she was a bad person (perhaps even sinful) and her trauma cognitions of self-blame persisted. That is, she still thought she was to blame, at least in part. Given that these thoughts were still negatively affecting her emotions she needed to explore what to do that could help her cope and continue to improve her associated depressed feelings (action plan).

Step 5: Action Plan: The therapist had noted that she mentioned she felt guilty and that she also mentioned that she considered herself to be a sinful person. The therapist asked permission to explore this with her and Adriana said yes. The first question was, what did she mean by “sinful”? What were her beliefs regarding sins or forgiveness? Adriana shared that she was not sure she was forgivable by God or by the elders, or anyone else, because of what she had done (placing herself and others in dangerous situations through defiance of her mother and by her actions). She thought her actions had had severe negative consequences for herself, her family and for others. She might be unforgivable. She was eventually able to identify that she ultimately believes in a forgiving God. She left the session willing to consider that God could possibly forgive her and maybe she could forgive herself. In considering that possibility she also suggested that one way to reappraise the situation would be to not fully blame herself for being harmed and for what had happened, and to at least lend some responsibility for what had happened to those who intentionally harmed her. She also considered the difficult circumstances that had influenced her decisions.

In a subsequent session, Adriana reported that she had had another dream, that felt more like a vision. She was being spoken to by a person sitting in the dark on the couch of her room at night. At first, she was not sure who the person was, but she felt it might be her boyfriend who had been killed by gang members when he had tried to protect her from them. This was the first time she had disclosed anything about him in therapy, even though it had always been in her memory along with deep feelings of grief, guilt, and shame. In the dream, her deceased boyfriend told her not to forget him but to move on with her life in a positive direction and that he would protect her. He said he forgave her. She felt comforted. This was a pivotal moment for Adriana, in being willing to reappraise (reframe) her guilt in the context of reconciliation with someone who could only be reached in a spiritual context.

In therapy, she continued to look at her trauma cognitions about the world, herself, and others, which over time improved, and she was able to explore self-compassion, and forgiveness and consider how she could be more reconnected to elders, family, community. Adriana initially compartmentalized and even suppressed her posttraumatic thoughts, allowing temporary respites from mental stress that allowed her to function. The trusting relationship with her therapist allowed her to explore spiritual themes and their larger meaning and helped her develop awareness to her feelings and to her thoughts. Beyond that, it was important for her to reconcile her guilt and sense of disconnection from family and community. Her posttraumatic symptoms improved along with her overall posttraumatic cognitions, and in particular cognitions about herself and self-blame improved (See Table 3, ID 1001).

### ***Vignette 2: Prayer and compassion offering agency over anger***

Ricky (a pseudonym) is a 14-year-old male participant from Central America who reported experiencing memories about his mother’s drug use and physical abuse by his stepfather. He had witnessed his mother being physically and sexually assaulted by men. He fled to the US border as an unaccompanied minor to escape violence in his home and community. In the United States, he had a hard time getting along with peers, teachers or with his foster parents. He was described by adults as disrespectful, and particularly “not respectful of women”. He had frequently been in trouble at school, including once for brushing up against a girl at school who then reported him to the school principal.

Ricky expressed anger about the world. Despite having a very angry and disruptive demeanor, he was willing to come to therapy. At first, he was resistant to talking about himself. Slowly, he opened to the idea of doing simple exercises like mindful eating, and mindful walking practices. Along with the therapist, they were able to frame these as fun, interesting games. With his therapist, Ricky completed modules on the cognitive triangle (i.e., understanding the connections between thoughts, feelings, and actions) and common styles of thinking (posttraumatic cognitions). With the therapist, he explored his ways of coping. He shared that he relied very strongly on religion as support, and he had even considered becoming a minister.

In being invited to practice, mindfulness to thoughts, he expressed that he was unsure that he wanted to lend any awareness to “bad” thoughts. At first his faith served as a potential barrier because he believed letting bad thoughts into one’s consciousness was a spiritual weakness that could even invite evil. This served as a barrier to his doing cognitive restructuring in therapy, although he was able to do a few exercises weighing the pros and cons of holding onto his anger. He considered how the way he acted out his anger through rages and attacking others were dissonant with his goals to graduate high school and to go to college. Anger was serving as a barrier for him, and he felt some motivation for addressing it. For these situations, he created an action plan on how to become more aware of his anger in the moment, he considered the different ways he could think about and respond to anger provoking situations and weighed his best options for improving his outcomes. He also realized that he sometimes smoked cannabis and drank alcohol to overcome anger and other bad feelings. He began to be more open to the idea of becoming more aware of his thoughts and began to engage in mindfulness practice.

Over time, he was able to embrace more advanced mindfulness practice in MBCT-Dual, specifically the Loving Kindness Meditation, which involves mentally sending goodwill and kindness towards others by silently repeating a series of mantras, usually: *May they be safe, May they be happy, May they be healthy, May they live with ease*. In reflecting on this meditation, Ricky decided to disclose that praying for his mother who was still living back in his home country and was struggling with substance abuse problems, was helpful for him. He shared that as he worked in therapy, he realized that praying for her made him feel better and was a way of reappraising his traumatic memories and his anger about how she had neglected him. Prayer offered him an opportunity to see his mother not simply as “all bad”, but also as someone deserving of prayer and maybe even forgiveness. That also presented the opportunity for him to consider that he may not be a bad person either (cognitions about self). His trauma cognitions shifted from the extreme, individuals are either all bad or all good, to the idea that someone can be ill or addicted and make bad decisions but still be deserving of compassion. Thus, his persistent feelings of anger towards his mother, and towards himself and more general perceptions of others (cognitions about the world), began to shift. This was a critical turning point in therapy. He began to feel self-mastery in navigating his feelings and behaviors at school and with others. His angry thoughts, especially his posttraumatic cognitions about the world, and his PTSD symptoms began to improve (See ID 1005, see Table 3).

### ***Vignette 3: Prayer as a Comforting Response to Posttraumatic Cognitions and Worries***

Martin (pseudonym) is a 15-year-old Salvadorean who enjoys using his creativity and music as a coping strategy. He often came to his therapy sessions with a notebook filled with lyrics for rap songs he had written to share with his therapist. He even performed rap songs in her office; others were meant to be sung with danceable Latin rhythms. His therapist encouraged him to keep working on his songs because it became evident that through the music, he was memorializing experiences of violence and traumatic loss (e.g., being shot at by gangs, death of friends, missing his family). Many of the themes in his songs included references to God’s protections and the importance of living a good life. When asked about these themes he shared that his faith in God is central to him. Martin worked well with exploring traumatic events in his life and building awareness of his thoughts and feelings through mindful practice. For example, he became aware that he had constant thoughts that he had abandoned his family, that they were in danger and did not have enough food, and it was in part his fault (cognitions about self-blame). He admitted that these guilt-ridden thoughts and worries sometimes made him unable to eat and made him feel very sad. He revealed that in the past he was unaware of the thoughts that were making him sad, and he sometimes smoked cannabis to improve his mood and appetite. Mindfulness practice made him aware of how his thoughts were making him feel, that he could safely explore these thoughts and that he had an opportunity to respond differently. He also began to explore how other experiences and events were impacting his thoughts about himself and others. He chose to use prayer as one of his coping strategies for his action plan for self-care (Step 5 CR). He expressed that praying helped him manage his worries about his family back in his country. Prayer offered relief because it was the way to “offer up” his worries to God, and request God’s protection for himself and his family. Prayer was comforting for him. The combination of practicing mindfulness, engaging in prayer, and creating music helped him develop agency for managing difficult feelings and improved his ability to compassionately explore and reframe his thoughts. He became more hopeful about the future and his own abilities. His depression symptoms improved. He stopped smoking cannabis. (See Table 3, ID 1007).

## Discussion

We tested the feasibility and implementation of MBCT-Dual in a small sample of traumatized unaccompanied immigrant children, a population for which there is minimal evidence-based research of treatments for PTSD. Our findings suggest that coaching on the use of adaptive coping, including exploring the possible role of spirituality-religiosity, can be helpful when integrated into cognitive therapy and mindfulness practice with unaccompanied immigrant children, especially if these can optimize culturally and spiritually congruent ways of understanding trauma and overcoming adversity. The children participating in this study had multiple traumatic experiences and all met criteria for PTSD at baseline. Our results show significant reductions in PTSD symptoms and in total negative posttraumatic cognitions at posttest for both the UIC and U.S.-born groups after receiving at least six sessions of a 12-session therapy. However, the differences in scores reflect a larger effect size for both symptom reduction and total cognitions for UIC compared to U.S.-born.

Our results demonstrate that evidence-based culturally responsive treatments are effective with UIC and are comparable with research with unaccompanied immigrant children conducted in European host countries (Rogers et al., 2018) using CBT and narrative therapy models (Unterhitzenberger et al., 2015). As noted earlier in this manuscript, religiosity and spirituality are constructs that can emerge in delivering cognitive therapies. For example, Adriana’s story includes meanings about community, ancestors and belonging as well as spiritual expressions of these that related to how she thought about herself and others. The treatment modifications for integrating religiosity and spirituality were feasible, required minimal adaptation to MBCT-Dual, and we encountered minimal difficulty in delivering the study intervention with UIC. The five-steps of cognitive restructuring and mindfulness practices were delivered with fidelity similarly across the two study groups. Step 5, Create an Action Plan, demonstrated unaccompanied immigrant children’s particular use of spirituality-religiosity for coping and problem-solving strategies.

### Implications for Interventions

Extending beyond the use of cognitive restructuring and including the opportunity to create an action plan (Step 5 of cognitive restructuring) is helpful in promoting the use of adaptive coping strategies including spirituality and religiosity. We found that negative posttraumatic cognitions about self (including self-blame) and about the world were significantly improved for unaccompanied child study participants and often included exploring, and in some cases reappraising, cognitions that were related to religious or spiritual beliefs. This was important for addressing negative cognitions that intersected with spiritually informed ways of understanding the world, self, and relationships with others. The composite vignettes illustrate some of the ways that spiritual and religious themes were explored in this way and integrated into MBCT-Dual.

Even though MBCT-Dual seemed helpful for the UIC in this study, we cannot state conclusions for the whole population of unaccompanied immigrant children, which consists of many different nationalities, ages, and diverse cultures. Yet a striking result from the pilot study (Fortuna et al., 2018), and the present analysis, is that all the UIC completed treatment. This suggests having access to treatment with a bilingual bicultural therapist who provided a connection to their Latinx native culture, including spiritual-religious aspects, may be an important engagement factor for children with disrupted family and community connections, and may help to engage UICs in cognitive behavioral treatments. Further research can contribute to understanding what additional domains of religiosity and spirituality can be most helpful, e.g., exploring religious beliefs for decision making or for self-compassion.

There were challenges encountered in delivering the therapy, some which are described in our case vignettes. Difficulties in successfully engaging youth in regular session attendance was one. Access to services, facilitated by dedicated case managers and foster parents, was highly important for successfully engaging UIC. For the non-UIC study participants who did not have an involved and committed adult, it was much more difficult to engage them and deliver the therapy. The first few sessions were important for orienting the youth to mindfulness practices, which was a foreign concept for most. Some perceived mindfulness as meditation, or that the goal was to make one’s mind “blank”, which makes one vulnerable to “evil influences” and thus against Christianity. The simpler mindfulness practices such as “walking mindfulness” helped youth experience mindfulness as a focusing practice rather than a religious one. The five-steps of cognitive restructuring were not intuitive for many UIC and for many “thinking about your thoughts” seemed unproductive, silly, and even potentially harmful and dysregulating. Experiencing how awareness to thoughts and behaviors provides an opportunity for change and healing helped with this initial barrier, as did inviting youth to share their spiritual-religious and other cultural conceptualization of problems, events, and coping.

### Limitations and Future Directions for Research

Several limitations should be considered. First, the statistical results cannot be generalized due to the small sample size. In addition, six cases can hardly represent the heterogeneity of the group of unaccompanied immigrant children, even for the population from Guatemala, Honduras, and El Salvador. Given the pilot nature of our study, we did not include a control group. Hence, our results do not permit any conclusions about comparisons to spontaneous improvement, the effect of time, attention, or alternative treatments. It cannot be ruled out that simply the fact of receiving more attention and having regular appointments with a bilingual caring professional could be sufficient to reduce symptoms in our patient group. Finally, since we do not report any follow-up data, the long-term treatment success, especially in situations of recurring adversity remains to be seen. In fact, a previous study showed re-emergence of severe PTSD symptoms in unaccompanied immigrant children who experience stressors such as being denied asylum (Unterhitzenberger et al., 2019). Future research should examine the long-term outcomes for cognitive therapy approaches that integrates spirituality and culturally focused strengths as part of coping, especially given chronic stressful exposures in this population.

## Conclusions

Our results are promising: MBCT-Dual is acceptable and feasible for unaccompanied immigrant children with PTSD. Future research should focus on controlled trials with sufficient follow-up to understand the efficacy, maintenance, and generalizability of results. Our findings suggest that mindfulness-based cognitive therapy can be helpful in improving negative posttraumatic cognitions in UIC, especially negative cognitions about the world. Negative posttraumatic cognitions about living in an unsafe, unpredictable, and harsh world were highly prevalent for the UIC participants and only point to the potency of traumatic environments and experiences to create elevated and chronic distress in unaccompanied immigrant children. We need to not only identify effective treatments for this population but also work towards providing them with safe, culturally responsive, trauma informed, developmentally appropriate care and equitable environments free of marginalization and psychological harm. Therapy models that honor belief systems, cultural and spiritual contexts can offer an opportunity for implementing effective, and acceptable interventions for posttraumatic healing by unaccompanied immigrant children.

## Previous presentation

This work has not previously been published
